# *In-situ* analysis and monitoring of antiscalant polyaspartate by mobile fluorescence spectroscopy

**DOI:** 10.1016/j.mex.2025.103488

**Published:** 2025-07-04

**Authors:** Michael Pettauer, Albrecht Leis, Walter Gotschy, Ronny Boch, Lukas Hasenhüttl, Martin Dietzel

**Affiliations:** a Graz University of Technology, Institute of Applied Geosciences, Rechbauerstrasse 12, 8010 Graz, Austria; b JR-AquaConSol GmbH, Steyrergasse 21, 8010 Graz, Austria; c Dr. Walter Gotschy, Ingenieur- und Sachverständigenbüro für Optik, Physik und Tracertechnik, Waidach 116, 5021 Adnet, Austria

**Keywords:** Green inhibitors, Polyaspartic acid, Scaling forensics, Carbonates, CaCO_3_, Nucleation, Precipitation, Tunnel drainage, Pipelines, Water treatment

## Abstract

Monitoring and controlling antiscalants, such as polyaspartate (PASP), in technical systems is essential to prevent scale formation and optimize inhibitor application. We developed a ***F**luorescence-based **I**n-situ **P**olya**s**partate **A**nalysis (FIPSA)* using a portable fluorometer equipped with optical fiber sensors for real-time PASP detection in various environmental and industrial settings. We quantified the impact of solution matrix effects, including pH, temperature, ionic strength, and turbidity, on fluorescence signals, emphasizing the need for on-site calibration. The FIPSA method enhances process efficiency by simplifying data acquisition for the planning and implementation of hardness stabilization systems and enabling dynamic PASP dosing adjustments for optimized inhibitor application during system operation (e.g. tunnel drainage, wells, pipelines, industry).

The FIPSA method

• enables real-time PASP concentration measurement with a detection limit of ≈1 mg/L
• is adaptable to different PASP formulations and water matrices
• reduces dependence on time-delayed laboratory analyses

## Graphical abstract

**Figure:**
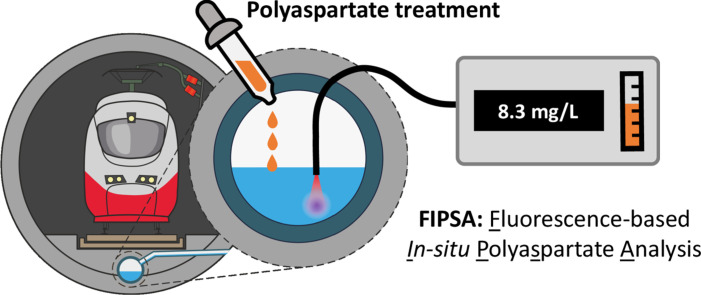

**Specifications table**

**Table:** 

**Subject area**	Chemical Engineering
**More specific subject area**	geochemical forensics, environmental monitoring, tracer tests, water-rock interaction, water quality assessment, scaling, water production and drainage, green inhibitors, water treatment
**Name of your method**	***F**luorescence-based **I**n-situ **P**olya**s**partate **A**nalysis (FIPSA)*
**Name and reference of original method**	
**Resource availability**	Mobile fluorometer with UV-source, combined quartz/PMMA optical fibers and software: https://gotschy.at/lichtleiter-fluorometer/

## Background

Polyaspartate (PASP) and its derivatives are ecofriendly, biodegradable and water-soluble chemicals used as antiscalant agent in order to delay, reduce, or even prevent the unwanted precipitation of solids from aqueous solutions (“scaling”). PASP can also modify the nucleation and growth conditions of various precipitates (e.g., CaCO_3_, CaSO_4_, BaSO_4_) from aqueous media, e.g. changing the crystal size and morphology of the solids and inhibiting their agglomeration, thereby also weakening their adherence to surfaces [[1], [2], [3], [4], [5]]. PASP is widely utilized in various industrial applications, including reverse osmosis and nanofiltration systems, geothermal and oilfield wells, and, more recently, drainage water treatment in railway and road tunnels to prevent clogging of the drainage pipes [[6], [7], [8]]. In the latter case, PASP demonstrates excellent inhibitory properties even at low concentrations of 2 to 10 mg/L, although the dosage needs to be adjusted and controlled to achieve optimal process conditions for a site-specific application [2]. Thus, the accurate and often spatially resolved (flow route) measurement of PASP concentration in operation is essential to assure cost-effective and technically feasible conditions of the inhibitor application.

Analytical methods regarding PASP include ¹H and ¹³C NMR spectroscopy, gel permeation chromatography, isotachophoresis as well as precipitation and polyelectrolyte titration. These methods provide valuable structural and/or concentration data but are generally less practical for field use. Their applications require sophisticated equipment, controlled conditions during analyses, and/or time-intensive preparations/analyses, thus being rather suitable for laboratory-based measurements. Detailed descriptions of these analytical techniques, including their principles, advantages, and limitations, can be found in Ullmann’s Encyclopedia of Industrial Chemistry: Polyaspartates and polysuccinimide [3]. Fluorescence spectroscopy is one of the most efficient and practical methods for measuring PASP concentrations. Due to the presence of chromophoric structures in thermally synthesized PASP, it can be excited by UV light (with a maximum at 336 nm) and emits light at approximately 411 nm [3,[9], [10], [11]]. Accordingly, after sampling (e.g. in the field) the PASP concentration is conventionally analysed using state-of-the-art lab-based fluorescence spectroscopy. The information obtained, however, is often limited based on the non-permanent and time-shifted results of the laboratory analyses [8,12]. For the latter need, we have developed an *in-situ* PASP fluorescence technique by using a robust, portable fluorescence spectrometer equipped with optical fiber cables and coupled with a data storage unit and analytical software, i.e. designed for *in-situ* and continuous (time-resolved) measurement of PASP concentration in the field (e.g. tunnel drainage, well, pipeline) or within a process line. On-site calibration yields in a lower limit of PASP concentration to be analysed of about 1 mg/L and with the capability to measure concentrations up to 5000 mg/L.

Various commercially available PASP products have been tested so far, along with critical assessments of solution matrix effects such as the prevailing pH, temperature, ionic strength and chemical composition, as well as turbidity. The herein presented ***F**luorescence-based **I**n-situ **P**olya**s**partate **A**nalysis* (FIPSA) is well-suited for targeted measurements, continuous monitoring and optimizing feedback regulation of PASP concentration in a wide range of environmental settings and technical processes.

## Method details

### Device adaption for measuring PASP

The basic functionality of a fiberoptic fluorometer is described elsewhere [[9], [10], [11],^13^]. A major technical modification of the fluorometer device [14] to become suitable for the present application consists in extending the wavelength range to include UV for the excitation of PASP. This required the utilization of a compact UV light source, as well as the use of optical fibers made of quartz instead of the commonly used optical fibers made of e.g. polymethyl methacrylate (PMMA) as the light transmission of the latter is largely limited to the visible spectral range of 400 to 700 nm. The wavelengths for excitation (Ex) and emission (Em) of PASP are given in the literature as Ex_max_ = 336 nm and Em_max_ = 411 nm [3]. An UV-LED in combination with an optical filter results in a central wavelength of 340 nm and a half-width of 10 nm. Since the costs for quartz light guides with an appropriate diameter and length are typically very high, a hybrid solution consisting of quartz and plastic light guides was chosen for the mobile sensors. The herein presented optical fiber consists of two quartz fibers and three PMMA fibers with a diameter of 1 mm each and a length of 5 m. The quartz fibers transport the UV excitation light at 340/10 nm, the PMMA plastic fibers transfer the fluorescent light of the dye in the range of approximately 400 to 540 nm. The transmission of the PMMA fibers is sufficient for the above-mentioned wavelength of the polyaspartate fluorescent light. The sensor initially outputs a signal with a maximum of 4095 mV at a resolution of 1 mV. When this limit is exceeded, the sensor reduces its sensitivity by a factor of five, allowing measurements up to 20475 mV with a reduced resolution of 5 mV.

### Device setup and usage

**Fig. 1:**
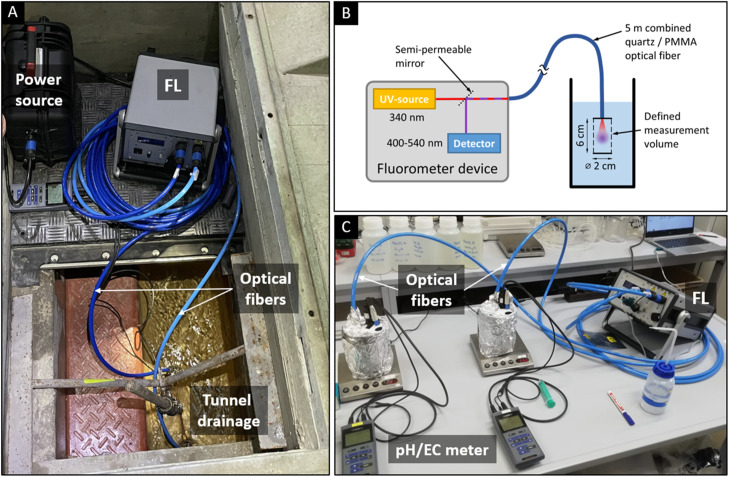
A: Field application of the mobile fluorometer for simultaneous measurement of PASP and Uranine. B: Schematic sketch of the fiberoptic fluorometer adapted for the measurement of PASP with an UV-source (LED). C: Usage of the FL-device in the lab with parallel measurement of the pH and electric conductivity (EC).

Fig. 1A shows a photograph of the mobile device for *in-situ* measurement of the PASP concentration within a drainage system of a railway tunnel. In this specific case a double tracer method was applied with continuous flow application of a combined PASP/Uranine mixture, therefore two separate optical fibers where used with a UV-light source and with a blue laser. Fig. 1B shows a schematic sketch of the mobile fluorometer device with its main components for FIPSA. In Fig. 1C the setup is shown for a laboratory application.

### Calibration

About 10 titration steps with a 1 mL pipette are recommended, using a pre-diluted PASP solution (diluted from original 40 wt. % stock solution) in order to achieve the targeted concentration range in a total volume of 1 L matrix water/solution. The titration is executed during constantly stirring for fluid homogenization near the sensor head. For minimization of light scattering during the calibration process an opaque container has to be used.

## Method validation

In the following section data is presented using 3 different and commercially available PASP products with groundwater and *pure* (Merck Milli-Q Type 1) water for dissolution. Calibration curves were generated, pH and temperature effects on the fluorescence signal of PASP were checked and further turbidity/adsorption was investigated using calcite (CaCO_3_) and bentonite powder. The measurements were conducted at 20 ± 1 °C and the temperature effect on the fluorescence signal was evaluated between 5 and 30 °C. The error bars in the diagrams represent a 2 Ϭ confidence interval (95.4 %).

For comparison lab-based fluorescence spectroscopic analyses of PASP were performed using a spectrofluorometer (Jasco FP-6500) with a standard quartz cuvette. All fluorescence measurements were carried out by the synchronous scan technique in constant-wavelength mode with a wavelength offset of 100 nm between excitation and emission. At these conditions, the PASP peak in the resulting synchronous fluorescence spectrum appears at approximately 415 nm. The intensity of the measured fluorescence signal is proportional to the concentration of aqueous PASP. The recorded fluorescence intensities were converted into concentration values by calibration. When applying the particularly sensitive synchronous scan technique, the detection limit for PASP is 0.15 mg/L, and the measurement uncertainty (1Ϭ) is approximately ±0.1 mg/L.

### PASP products

PASP-B, PASP-C and PASP-S are commercially available sodium polyaspartate solutions containing ≈ 40 wt. % of solids at densities ranging between 1.2 and 1.3 g/cm³. The average molar weight of the polyaspartate products ranges from 1000 to 5000 g/mol. Considering monomeric sodium aspartate C_4_H_4_NO_3_Na (137.07 g/mol), the average chain lengths of the polymers range between 7 and 36 monomeric units. All of the PASP products are random mixtures of *D*- and *L*-aspartate units [15]. Table 1 shows the PASP products used and their main characteristics, which are provided by the manufacturers. The names of the manufacturers are treated confidentially, but can be provided by the authors on request.

**Table 1:** List of selected PASP products used for method validation.

Abbreviation	Manufacturer	Agent	Ave. mol. wt.	Density	pH	Active agent
			*g/mol*	*g/cm³, 20* °*C*	*(10* g/L *solution)*	*wt. %*
PASP-B	B*******	Polyaspartic acid sodium salt	2000–3000	1.3	9.5–10.5	40
PASP-C	C*******	Polyaspartic acid sodium salt	1000	1.25	9.2	40
PASP-S	S*******	Polyaspartic acid sodium salt	1000–5000	1.2	9–11	40

### Calibration, detection limits, and precision

**Fig. 2:**
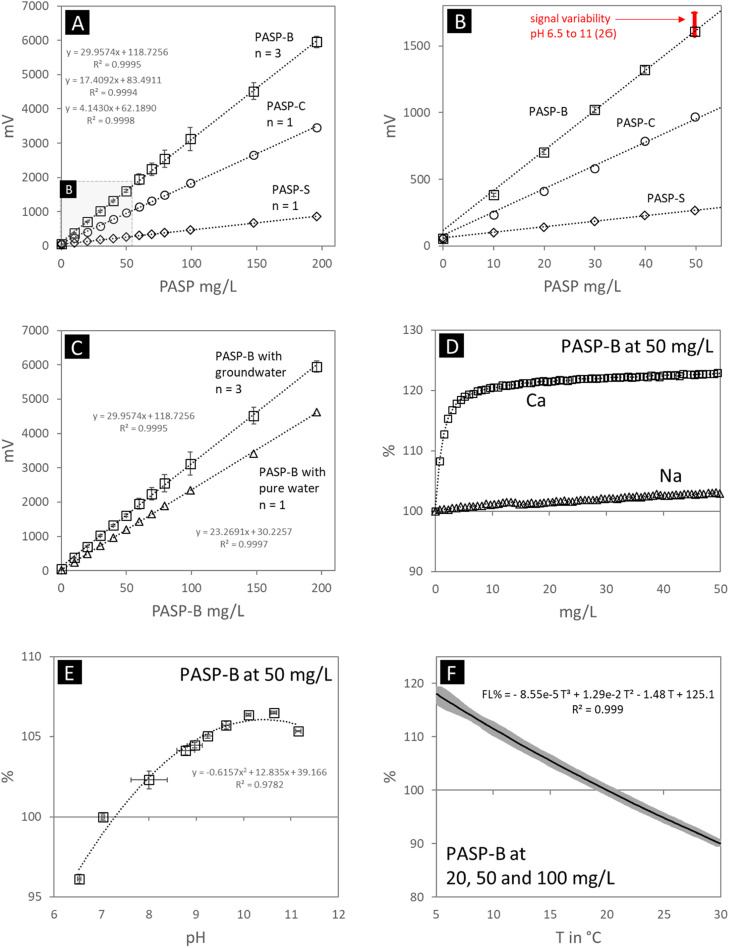
A: Calibration of 3 different PASP products with groundwater matrix from 10 to 200 mg/L. B: Detail of calibration from 10 to 50 mg/L including pH-dependent signal variability (pH from 6.5. to 11). C: Calibration of PASP-B with groundwater matrix versus pure water. D: Effect of Ca or Na concentration on the fluorescence signal at a PASP-B concentration of 50 mg/L. E: Variability of the fluorescence signal within the pH range of 6.5 to 11 using PASP-B at 50 mg/L. F: Dependency of the fluorescence signal on temperature, averaged across PASP-B concentrations of 20, 50, and 100 mg/L in groundwater. Error bars and grey area depicts 2Ϭ confidence interval.

For validation purpose we used a pre-diluted PASP solution of 10 mg/mL with titration steps of 1 mL each for the concentrations of 10, 20, 30, 40, 50, 60, 70, 80, 100, 150 and 200 mg/L of PASP (Fig. 2A). PASP fluorescence depends on the amount of chromophoric structures within the polymer chain, thus being related to the manufacturing process, the precursor material used and the resulting average molecular weight of the PASP (polymer length) [3,15]. PASP-B shows the highest fluorescence followed by PASP-C and PASP-S with the lowest.

The triplicate of PASP-B displays an excellent reproducibility of the signal especially below 50 mg/L and about 2000 mV, where the error bars are within the symbol size in Fig. 2B The highest deviation was observed between 2500 and 4000 mV, which may be related to the sensitivity switch of the sensor at 4095 mV.

The limit of detection (LOD = 3.3 ∙ Ϭ / slope) and the limit of quantification (LOQ = 10 ∙ Ϭ / slope) for PASP-B were calculated using the calibration curves of triplicates for the concentrations 10 to 50 mg/L: PASP-B_LOD_ = 1.3 mg/L and PASP-B_LOQ_ = 4.0 mg/L.

The method’s analytical precision was assessed by the relative standard deviation (RSD) across the effective measurement range (0–6500 mV) using PASP-B. For signal intensities between 0 and 2000 mV (corresponding to 0–50 mg/L PASP-B), the RSD was consistently below ±2 %. In the range of 2000–4095 mV (50–100 mg/L PASP-B), the RSD remained below ±5 %. Beyond 4095 mV, the sensor automatically reduces its sensitivity by a factor of 5 to extend the dynamic range. Within the evaluated post-switch range (4095–6500 mV, corresponding to 100–200 mg/L PASP-B), the RSD returned to below ±2 %, indicating restored precision after the sensitivity adjustment. At higher signal intensities, approaching the upper limit of 20475 mV, the error is expected to gradually increase again toward ±5 %.

### Matrix effects

We used a typical calcium-bicarbonate dominated groundwater (see chemical composition in Table 2) as well as *pure* water for the evaluation of the effect of ionic strength on the sensor (mV) signal. The mV signal is about 25 % lower for the *pure* water compared to the groundwater (Fig. 2C). This effect is caused by the matrix influence of aqueous Ca ions, which is approximately 80 mg/L in the groundwater. A 50 mg/L solution of PASP-B in pure water, with the constant addition of CaCl₂, results in an increase of the mV signal by 20 to 25 % within the first 5 to 10 mg/L Ca ions added (Fig. 2D), which remains far below the typical Ca concentrations observed in environments where CaCO₃ scaling is critical. In contrast, the addition of NaCl to the same solution led to only ≈ 3 % increase when reaching a Na ion concentration of 50 mg/L (Fig. 2D). Nevertheless, considering the matrix as a key influencing factor is essential, it is highly recommended to use the original matrix (water) to calibrate the fluorometer on-site. Subsequent calibration with the collected solution is also possible.

**Table 2:** Electric conductivity (EC), pH and chemical composition of the groundwater used for validation.

	pH	EC at 25 °C	Na^+^	*K*^+^	Mg^2+^	Ca^2+^	Cl^-^	NO_3_^-^	SO_4_^2-^	HCO_3_^-^
		µS/cm	mg/L	mg/L	mg/L	mg/L	mg/L	mg/L	mg/L	mg/L
Groundwater	7.5	529	7	2	17	80	11	7	36	254

The influence of variable pH on PASP analysis by FIPSA was assessed using groundwater with PASP-B addition at a concentration level of 50 mg/L. This solution was titrated with 0.5 M HCl or NaOH to yield a pH range from 6.5 to 11, which is the common range of groundwater, surface water, and solutions gained from concrete interaction in technical/constructional settings [8,[16], [17], [18]]. The fluorescence signal increased from pH 6.5 with a maximum at about 10.5 and decreased slightly by reaching pH 11 (Fig. 2E). This systematic change of the fluorescence signal is caused by the pH dependent dominance of protonation states of aqueous polyaspartate species [19]. The relative deviation of the signal within the above pH range is about 11 % and is emphasized as red error bar in Fig. 2B (with 100 % signal at a pH of 7).

**Fig. 3:**
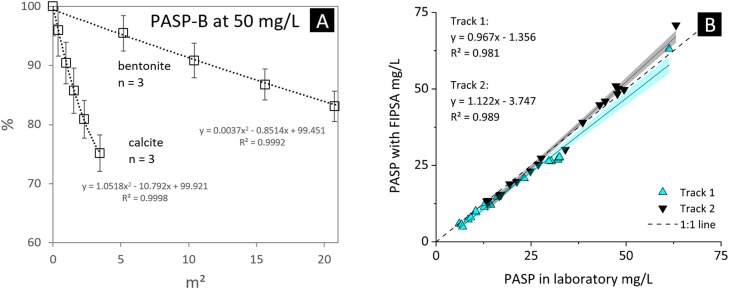
A: Partial signal loss depending on turbidity and PASP adsorption phenomena. B: Correlation of PASP concentrations between laboratory and field measurements. The dotted line represents the 1:1 reference, where values from both methods would be identical. Regression lines and confidence intervals (95 %) are shown for measurements in drainage pipes of the two separate tunnel tubes: Track 1 (cyan) and Track 2 (black).

The evaluation of adsorption and/or turbidity effects on the fluorescence signal was carried out – in analogy to the pH effect – using the calcium-bicarbonate dominated groundwater at a concentration of 50 mg/L PASP-B. Therefore, bentonite (B15; [20]) and calcite (CaCO_3_, Roth ≥99 % p.a.) powder with specific surface areas of 101 and 3.75 m²/g, respectively, were suspended in the groundwater (Fig. 3A). As the powdered material is gradually added to the groundwater, the fluorescence signal decreases. For bentonite, the dominant effect is likely turbidity, which scatters light and reduces the amount of fluorescent signal reaching the PMMA optical fiber. In contrast, the effect of calcite powder is significantly more pronounced relative to its absolute surface area. This suggests that the primary mechanism for calcite is the adsorption of PASP onto the crystal surfaces, effectively reducing the active PASP concentration in solution and decreasing the fluorescence signal more strongly than bentonite. Consequently, FIPSA analyses in suspensions must account for and quantify both potential background signals and partial PASP adsorption.

### Temperature effect

The effect of temperature on fluorescence intensity is well known, typically leading to a decrease as temperature increases (Fig. 2F) [9,21]. To evaluate this effect, groundwater containing PASP-B at concentrations of 20, 50, and 100 mg/L was measured within a temperature range of 5 to 30 °C. The results were averaged and expressed as a percentage relative to the fluorescence intensity (FL %) at 20 °C (100 %). The observed dependency is nearly linear within this temperature range and can be described by a third-order polynomial function as follows (T in °C):

FL % = – 8.55 × 10^–5^ × T^3^ + 1.29 × 10^–2^ × T^-2^ – 1.48 × T + 125.1

### Comparison of FIPSA with laboratory measurements

*In-situ* measurements can be influenced by specific environmental factors, such as fluctuating temperatures within the drainage system or incident light, whereas laboratory analyses are conducted under controlled conditions, being an appropriate reference for validation. This was particularly relevant for validating PASP measurements, since no prior reference data were available for *in-situ* PASP analysis.

In a field campaign the FIPSA method was applied for measuring the concentration of PASP addition in the tunnel drainage system of the Koralm railway tunnel (Austria; Fig. 1A) [16]. The comparison of the PASP concentrations from the FIPSA methods versus the laboratory measurements are displayed in Fig. 3B. As demonstrated, there is a strong agreement between the two analytic approaches. The statistical correlation coefficient (R² ≥ 0.98) indicates a very high degree of consistency between the datasets. This confirms that both methods measure PASP concentrations with minimal variation.

## Limitations

Compared to conventional laboratory devices, the FIPSA method shows lower sensitivity and therefore a higher detection limit, e.g., approximately 1 mg/L for PASP-B. Background fluorescence may potentially be caused by organic compounds in the analysed medium, compromising the LOD as well as variations in fluorescence intensity among different PASP products. Due to herein developed FIPSA design a minimum sample volume of approximately 100 mL is required. Additionally, factors such as case-specific turbidity, adsorption phenomena, and laminar versus turbulent flow conditions can influence the fluorescence signal, potentially affecting measurement accuracy, precision and reliability.

## Ethics statements

This study did not involve human subjects or animals or involved data collected from social media platforms and therefore did not require ethical review.

## CRediT authorship contribution statement

**Michael Pettauer:** Conceptualization, Investigation, Formal analysis, Writing – original draft, Visualization. **Albrecht Leis:** Conceptualization, Methodology, Writing – review & editing, Project administration. **Walter Gotschy:** Methodology, Writing – original draft. **Ronny Boch:** Conceptualization, Writing – review & editing. **Lukas Hasenhüttl:** Investigation. **Martin Dietzel:** Conceptualization, Resources, Writing – review & editing, Project administration.

## Declaration of competing interest

The authors declare that they have no known competing financial interests or personal relationships that could have appeared to influence the work reported in this paper.

## Data Availability

Data will be made available on request.
